# Epidemiologic and Molecular Analysis of Human Tularemia, United States, 1964–2004

**DOI:** 10.3201/eid1207.051504

**Published:** 2006-07

**Authors:** J. Erin Staples, Kristy A. Kubota, Linda G. Chalcraft, Paul S. Mead, Jeannine M. Petersen

**Affiliations:** * Centers for Disease Control and Prevention, Fort Collins, Colorado, USA;; † Centers for Disease Control and Prevention, Atlanta, Georgia, USA

**Keywords:** Tularemia, Francisella tularensis, Epidemiology, Pulsed-Field Gel Electrophoresis, Ecology, Vector-borne Diseases, United States, Research

## Abstract

Tularemia in the United States is caused by 2 subspecies of *Francisella tularensis*, subspecies *tularensis* (type A) and subspecies *holarctica* (type B). We compared clinical and demographic features of human tularemia cases from 1964 to 2004 from 39 states in which an isolate was recovered and subtyped. Our data indicate that type A and type B infections differ with respect to affected populations, anatomic site of isolation, and geographic distribution. Molecular subtyping with pulsed-field gel electrophoresis further defined 2 subpopulations of type A (type A-east and type A-west) that differ with respect to geographic distribution, disease outcome, and transmission. Our data suggest that type A-west infections are less severe than either type B or type A-east infections. Through a combined epidemiologic and molecular approach to human cases of tularemia, we provide new insights into the disease for future investigation.

## Abstract 2

Distinct subpopulations of *F. tularensis* differ in their clinical manifestations, geographic distribution, and likely modes of transmission.

Tularemia is a zoonotic disease caused by the gram-negative bacterium *Francisella tularensis* (*1*). Transmission occurs through arthropod bites (especially ticks and deerflies), ingestion of contaminated food or water, inhalation of contaminated aerosols, and handling of infected animal tissues. Human illness usually takes 1 of several clinical forms. The most common is ulceroglandular tularemia; more serious forms include pneumonic, typhoidal, and meningitic tularemia.

Nearly all human cases of tularemia in the United States are caused by *F. tularensis* subspecies *tularensis* (type A) or *F. tularensis* subspecies *holarctica*, (type B) (*2*). Type A and B isolates can be differentiated on the basis of glycerol fermentation, virulence in animal models, and by polymerase chain reaction (*3**–**5*). Recently, molecular assays have been developed to further discriminate within subspecies by using pulsed-field gel electrophoresis (PFGE), multiple-locus variable–number tandem repeat analysis (MLVA), whole-genome microarrays, and single nucleotide variations (*6**–**9*). In nature, the 2 subspecies are thought to be maintained in distinct but incompletely defined cycles (*10**–**12*).

Although both subspecies of *F. tularensis* cause human illness (*10*), the clinical and epidemiologic features of type A and type B infections have not been systematically compared for a substantial number of cases. Furthermore, the implications of other subgroupings, as defined by molecular techniques, are largely unknown. We identified to subspecies level all human *F. tularensis* isolates that were submitted to the Centers for Disease Control and Prevention (CDC) for a 40-year period and further subtyped a portion by PFGE. Our findings demonstrate distinct subpopulations of *F. tularensis* that differ in their clinical manifestations, geographic location, and likely modes of transmission.

## Methods

We analyzed all available *F. tularensis* isolates from humans (n = 316) recovered by or submitted to CDC by state and local health departments from 1964 through 2004. All work with *F. tularensis* cultures was performed in a biosafety level 3 (BSL-3) laboratory using BSL-3 safety precautions. Isolates were confirmed as *F. tularensis* by characteristic growth on agar and direct fluorescence antibody staining. Type A and type B isolates were differentiated by biochemical subtyping (glycerol fermentation) with the 96-well automated MicroLog MicroStation System with GN2 Microplates (Biolog Inc, Hayward, CA, USA) (*13*).

Demographic and clinical data on source patients were extracted from submission forms that accompanied the isolates. Extracted information included patient age and sex, date of disease onset, form of clinical disease, anatomic source of isolate, underlying illness, outcome, county where infected, and likely mode of transmission. Isolates received after 1990 (n = 155) were matched with patients reported through the National Electronic Telecommunication Surveillance System to extract county of exposure. Information was verified by contacting the reporting state health department.

PFGE subtyping was performed on a subset of isolates with a modified version of the PulseNet 1-day standardized protocol for subtyping foodborne pathogens (*14**,**15*). A total of 41 type A and 22 type B isolates were used. DNA-embedded agarose plugs were prepared and lysed under BSL-3 conditions. PFGE plugs were cut (2.0 mm) and digested with 40 U *Pme*I enzyme (New England Biolabs, Beverly, MA, USA) for 6 hours at 37°C under BSL-2 conditions. *Salmonella enterica* serotype Braenderup (H9812) was used as a reference standard, and DNA plugs were digested with 50 U *Xba*I enzyme (Roche Diagnostics, Indianapolis , IN, USA) for 3 hours at 37°C. Seakem agarose gels (1%) were prepared with 0.5× Tris-borate-EDTA buffer (Sigma, Saint Louis, MO, USA), and digested DNA plugs were loaded on the comb. Electrophoresis was performed with a CHEF Mapper (Bio-Rad, Hercules, CA, USA) with switch times of 1.79 to 10.71 s at 6V/cm for 17.5 h at 14°C. Gels were stained with ethidium bromide (1 mg/mL) and gel images captured by using a Gel Doc 1000 imager (Bio-Rad).

Analysis of PFGE gels was performed with BioNumerics software version 3.0 (Applied Maths BVBA, Sint-Martens-Latem, Belgium). Gels were normalized by using the *Salmonella* reference strain. A dendrogram was constructed with Dice similarity coefficients and unweighted pair group method with averages (UPGMA). Demographic and clinical data were analyzed with EpiInfo 2002 (CDC, Atlanta, GA, USA). To analyze categorical data and nonparametric tests for continuous data, χ^2^ was used.

## Results

**Figure 1:**
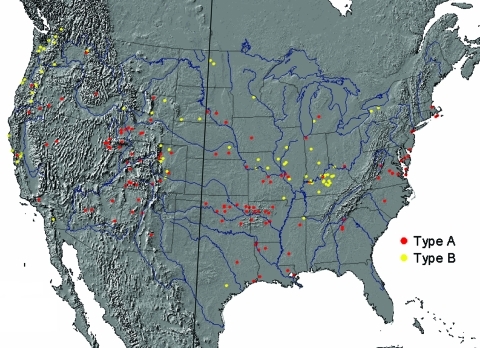
Geographic distribution for type A (red circles) and type B (yellow circles) *Francisella tularensis* isolates from humans, contiguous United States, 1964–2004. Each circle represents 1 isolate. Isolates were plotted randomly within the county of exposure. The 100th meridian is indicated by the black line transecting the United States. County of exposure was known for 198 (63%) isolates.

A total of 316 *F. tularensis* isolates from 39 states were available for analysis; 208 (66%) were type A, and 108 (34%) were type B (Table 1). Among the 10 states submitting at least 10 isolates, the distribution of the 2 subspecies was nonrandom (χ^2^ = 34, p <0.0001). Overall, the 2 subspecies segregated into several geographically distinct clusters (Figure 1). Most isolates on the eastern seaboard, in and around Arkansas and Oklahoma, and in the broad area from the Colorado Rockies west to the Sierra Nevada Mountains were type A. In contrast, most isolates from the northern Pacific Coast and along tributaries of the Mississippi River were type B.

**Table 1:** Number of type A and type B *Francisella tularensis* isolates, United States, 1964–2004*

State	Type A	Type B	Total
Alaska	2	0	2
Arizona	4	0	4
Arkansas	16	0	16
California	3	7	10
Colorado	19	8	27
Delaware	3	0	3
Georgia	5	1	6
Idaho	1	2	3
Illinois	2	6	8
Indiana	1	6	7
Iowa	2	1	3
Kansas	9	0	9
Kentucky	3	20	23
Louisiana	7	0	7
Maine	4	0	4
Maryland	5	0	5
Michigan	1	2	3
Minnesota	0	1	1
Mississippi	2	1	3
Missouri	12	8	20
Montana	0	1	1
Nebraska	6	1	7
Nevada	9	0	9
New Jersey	1	0	1
New Mexico	6	0	6
New York	5	3	8
North Carolina	4	0	4
North Dakota	3	5	8
Ohio	1	1	2
Oklahoma	14	1	15
Oregon	8	14	22
Pennsylvania	1	0	1
South Dakota	9	4	13
Tennessee	4	0	4
Texas	4	1	5
Utah	8	0	8
Virginia	9	1	10
Washington	0	8	8
Wyoming	13	3	16
Unknown	2	2	4
Total	208	108	316

*Submitted to the Centers for Disease Control and Prevention.

**Figure 2:**
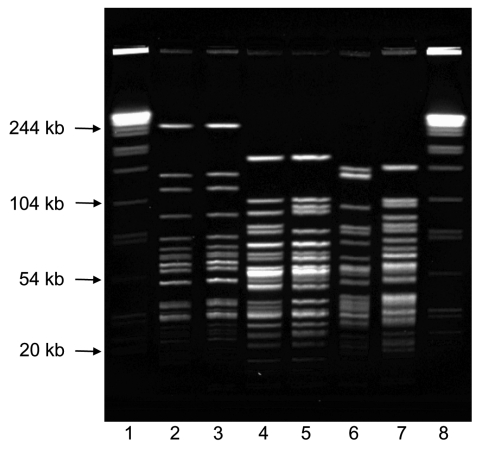
*Pme*I pulsed-field gel electrophoresis patterns for *Francisella tularensis* type A and type B. Lanes 1 and 8, *Salmonella enterica* serotype Braenderup standard; lane 2, Virginia 1997 type B; lane 3, Indiana 1999 type B; lane 4, New York 2004 type A-east; lane 5, Oklahoma 2001 type A-east; lane 6, Oregon 2004 type A-west; lane 7, California 2002 type A-west.

To further understand this geographic clustering, we developed a PFGE subtyping method *F. tularensis* using *Pme*I. PFGE differentiated both between type A and B strains and among type A strains (Figure 2). Electrophoresis conditions were optimized to resolve restriction fragments between 25 kb and 125 kb for both *F. tularensis* and the *Salmonella* reference strain. Reproducibility testing verified that no differences in PFGE patterns were observed between experiments.

**Figure 3:**
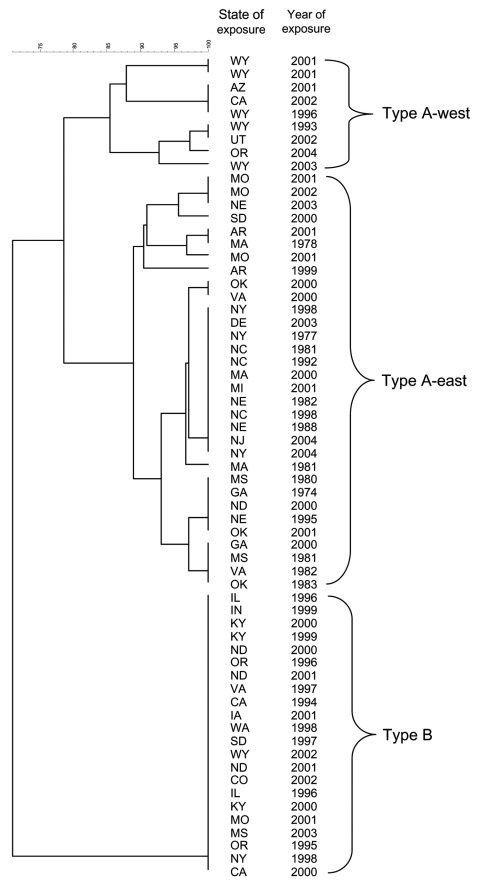
Dendrogram based on *Pme*I pulsed-field gel electrophoresis patterns of 41 *Francisella tularensis* type A and 22 type B isolates from humans. A 1.5% optimization and 1.5% tolerance were used to calculate Dice similarity coefficients. Isolate information is given with respect to exposure state and year.

Comparison of PFGE patterns for a subset of isolates (41 type A, 22 type B) from distinct locations (Figure 3) showed that all 22 type B isolates yielded the same PFGE pattern, which is consistent with previous data showing that type B strains exhibit little genetic diversity (*7*). In contrast, the type A isolates yielded PFGE patterns that fell into 2 main clusters (Figure 3; 32 type A-east, 9 type A-west). Isolates from 1 cluster came from states completely west of the 100th meridian (type A-west), while the remainder came from states transecting or east of the 100th meridian (type A-east, Figure 1). Based on the PFGE clustering, subsequent epidemiologic analysis was performed separately for type A isolates from the eastern (n = 133; type A-east) and western (n = 71; type A-west) contiguous United States.

Information on age and sex was known for 290 (92%) and 312 (99%) source patients, respectively. Males accounted for 75% (157/208) of type A and 72% (75/104) of type B infections. Patients with type A infections were significantly younger than patients with type B infections (median age 38 years vs. 50 years, p<0.01), and patients with type A-west infections were significantly younger than those with type A-east infections (median age 33 years vs. 44 years, p <0.02). An immunocompromising condition (e.g., malignancy, organ transplant, and HIV infection) was reported for 11 (10%) of 108 type B–infected patients, 6 (5%) of 133 type A-east–infected patients, and none (0%) of 68 type A-west–infected patients (p<0.01). For the 235 (74%) source patients for whom outcome was known, the overall case-fatality rate was 9% (20/235), with similar rates for infections caused by type A (9%, [15/161]) and type B (7%, 5/74) isolates. However, among type A infections, case-fatality rates differed markedly between type A-east (14% [15/106]) and type A-west (0% [0/55]) (p<0.002).

Information on anatomic source was available for 280 (89%) isolates. Overall, more than half of the isolates were recovered from lymph nodes and a quarter from blood (Table 2). While type A and type B isolates did not differ significantly with respect to anatomic source, significant differences were observed between type A-west and both type A-east (p<0.001) and type B (p<0.002) isolates. Type A-east and type B isolates were more likely than type A-west isolates to be recovered from blood and lung (Table 2), whereas type A-west isolates were more likely to be recovered from lymph nodes.

**Table 2:** Anatomic source of *Francisella tularensis* isolates, United States, 1964–2004

Site	Type A, no. (%)*	Type B, no. (%)	Type A-east, no. (%)	Type A-west, no. (%)
Blood	46 (25)	24 (24)	43 (36)	3 (5)
Cerebrospinal fluid	5 (3)	0 (0)	4(3)	1 (2)
Eye	2 (1)	5 (5)	1(1)	1 (2)
Lung	18 (10)	12 (12)	14 (12)	3 (5)
Lymph node	109 (60)	58 (59)	56 (48)	53 (87)

*One additional isolate was recovered from a penial exudate.

The clinical form of the disease was known for only 104 (33%) source patients. Ulceroglandular and glandular were the most commonly reported clinical forms of tularemia, accounting for 68 (65%) of 104 cases with information available. Other clinical syndromes included pneumonic (17%, 18/104), typhoidal (12%, 12/104), oculoglandular (4%, 4/104), meningitic (1%, 1/104) and pharyngeal (1%, 1/104) forms. Patients with typhoidal or pneumonic disease were generally older (median age 48 years and 53 years, respectively) than those with either glandular or ulceroglandular disease (median age 11 years and 37 years, respectively). Among the subset of patients for whom a clinical form of infection was reported, glandular or ulceroglandular disease was diagnosed more frequently in patients with type A infections (71% vs. 52% for type B) and pneumonic disease was diagnosed less frequently (12% vs. 30% for type B).

**Figure 4:**
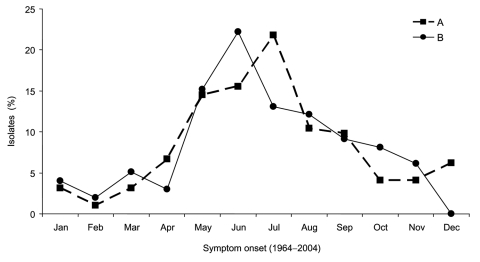
Seasonal distribution of type A and type B *Francisella tularensis* isolates from humans, United States, 1964–2004.

Among 292 source patients whose date of disease onset was known, 210 (72%) were infected in May through September; no difference in onset between type A and B infections was noted (Figure 4). A possible source of infections was reported for 133 (42%) of 316 patients. Direct animal contact accounted for 47 (47%) of 99 type A and 18 (53%) of 34 type B infections. Most type A infections were associated with exposure to either lagomorphs (53%, 25/47) or cats (30%, 14/47). Type B infections were most often associated with exposure to rodents (33%, 6/18) or cats (22%, 4/18); none were linked to lagomorph exposure. Arthropod bites accounted for 44 (44%) type A infections and 10 (29%) type B infections. Although the arthropod involved was not always identified, ticks were reported as the source for 21 (100%) of 21 type A-east, 9 (100%) of 9 type B, and 4 (44%) of 9 type A-west infections. Biting flies were only linked to type A-west infections and accounted for 5 (55%) of 9 type A-west infections attributed to a known arthropod bite. The remaining 8 (8%) type A and 6 (18%) type B infections were in patients with both animal and arthropod exposure or were attributed to environmental exposures such as landscaping.

## Discussion

Categorization of *F. tularensis* type A and type B was first proposed by Olsufiev et al. in 1959 (*16*). These 2 subspecies have been suggested to differ in their ecology and possibly their virulence for humans (*17*). Our study, which includes isolates obtained during a 40-year period, further refines these views. By combining PFGE subtyping of isolates with geographic data, we found evidence that human type A infections can be further divided into at least 2 distinct subgroups (type A-east and type A-west). In addition, our data suggest that type A-west infections are less severe than type B or type A-east infections.

The most notable finding of this investigation concerns fatality rates. The case-fatality rate for type A-east infections was 14%, compared with 7% for type B infections and 0% for type A-west infections. Type A-east and type B isolates were more likely to have been recovered from patients' lungs or blood, whereas type A-west isolates were more often isolated from lymph nodes. The apparent reduced invasiveness of type A-west strains may be explained by the younger age of the patients infected. However, a review of the literature shows that few, if any, tularemia deaths have been reported in the Rocky Mountain region (*18**–**21*), regardless of patient age. We hypothesize that the milder form of clinical disease associated with type A-west may be due to differences in virulence factors or perhaps infectious doses associated with differing modes of transmission.

Infections caused by type A-west, type A-east, and type B appear to occur in distinct geographic foci, suggestive of different ecologic niches. Type B infections cluster along major waterways, such as the upper Mississippi River, and in areas with high rainfall, such as coastal areas of the Pacific Northwest. Type A-west infections predominate in the arid region from the Rocky Mountains west to the Sierra Nevada Mountains. Type A-east infections occur in 2 main areas: 1) the central southeast states of Arkansas, Missouri, and Oklahoma and 2) along the Atlantic Coast, east of the Appalachians. The central southeast region is a major focus of human tularemia, accounting for 50% of all reported cases in the United States (*22*). The type A-east infections along the Atlantic Coast may be linked to the importation of rabbits from Arkansas, Oklahoma, Missouri, and Kansas to hunting clubs in Massachusetts, Pennsylvania, New Jersey, and Maryland in the 1920s and 1930s (*17**,**23*). Whether the geographic demarcation of type A-west and type A-east adheres strictly with the 100th meridian will require further PFGE analysis of more type A strains.

Animal studies have indicated that type A isolates are often associated with lagomorphs (rabbits and hares), while type B isolates are more often obtained from rodents (*12**,**17*). Consistent with these findings, our results show that human type A-east and type A-west infections were associated with exposure to lagomorphs, whereas human type B infections were associated with exposure to rodents. Both type A and type B infections were associated with exposure to cats. Ticks were implicated in transmission of both type A-east and type B infections to humans, whereas biting flies were only implicated in transmission of type A-west infections. The restriction of deerflies (*Chrysops* spp.) and associated human cases to western states was previously noted by Jellison (*24*).

This study is subject to several limitations, including record completeness, ascertainment bias, and number of *F. tularensis* isolates PFGE subtyped. Subspecies differentiation for *F. tularensis* has historically been dependent on the recovery of an isolate, and subtype information is not captured in the national disease reporting system. The overall case-fatality rate in this study was 9%, which is severalfold higher than the <2% previously reported (*25*). The higher rate suggests enhanced ascertainment of fatal cases. In addition, 36% of type A-east and 25% of type B isolates were recovered from blood, a much higher rate than previously reported. By analyzing only patients with culture-confirmed infections, we may have selected for patients with more fulminant disease.

Our results demonstrate that *Pme*I PFGE subtyping is useful for dividing type A isolates into geographically and clinically meaningful subgroups. Nineteen type A isolates analyzed by PFGE in this study (12 type A-east, 7 type A-west) were previously analyzed by MLVA and divided into 2 subpopulations, A.I and A.II (*26*). Subpopulations independently identified by the 2 methods are in complete agreement, which suggests that type A-west is analogous to A.II and type A-east is analogous to A.I. With training and interlaboratory validation, the PFGE method described here could be adopted by PulseNet laboratories throughout the country that use the standardized PulseNet PFGE protocol for foodborne pathogens (*27*). The PulseNet network is an existing laboratory infrastructure with all of the necessary equipment and software to perform, normalize, and compare PFGE patterns. PFGE subtyping of *F. tularensis* isolates would allow states to determine the potential geographic origins of tularemia cases and also share and compare their PFGE patterns within the PulseNet network.

Although type A is often referred to as the more virulent subspecies of *F. tularensis* and of greatest concern with respect to bioterrorism, our comparative analysis suggests that this view should be reevaluated. We found that human type A-west infections are markedly less severe than type B infections. Further studies are warranted to determine the basis of the clinical, geographic, and ecologic differences between infections caused by type B, type A-west, and type A-east.
